# Knockdown of long non-coding RNA ANRIL inhibits tumorigenesis in human gastric cancer cells via microRNA-99a-mediated down-regulation of BMI1

**DOI:** 10.1590/1414-431X20186839

**Published:** 2018-08-16

**Authors:** Pei Liu, Mingming Zhang, Qinghui Niu, Fengjuan Zhang, Yuling Yang, Xiangjun Jiang

**Affiliations:** 1 Department of Infectious Diseases, The Affiliated Hospital of Qingdao University, Qingdao, Shandong, China; 2 Department of Gastroenterology, Qingdao Municipal Hospital, Qingdao, Shandong, China

**Keywords:** Gastric cancer, lncRNA ANRIL, miR-99a, BMI1, Apoptotic pathway, Notch/mTOR pathway

## Abstract

Long non-coding RNA antisense non-coding RNA in the INK4 locus (ANRIL) has been reported to promote tumorigenesis via regulating microRNA (miR)-99a in gastric cancer cells. However, the role of each component involved in it is still not well understood. This study aimed to verify the role of ANRIL in gastric cancer as well as the underlying mechanisms. ANRIL levels in clinical gastric cancer tissues and cell lines were tested by qPCR. Effects of ANRIL silence on cell viability, migration and invasion, apoptosis, and miR-99a expression in MKN-45 and SGC-7901 cells were measured using CCK-8, Transwell assay, flow cytometry, and qPCR assays, respectively. Then, effects of miR-99a inhibition on ANRIL-silenced cells were evaluated. B-lymphoma Mo-MLV insertion region 1 (BMI1) expression, after abnormal expression of ANRIL and miR-99a, was determined. Finally, expression of key proteins in the apoptotic, Notch, and mTOR pathways was assessed. ANRIL level was elevated in gastric cancer tissues and cell lines. Knockdown of ANRIL suppressed cell viability, migration, and invasion, and increased apoptosis through up-regulating miR-99a. Furthermore, ANRIL silence down-regulated BMI1 via up-regulating miR-99a. BMI1 silence down-regulated Bcl-2 and key kinases in the Notch and mTOR pathways and up-regulated p16 and cleaved caspases. We verified the tumor suppressive effects of ANRIL knockdown in gastric cancer cells via crosstalk with miR-99a. Together, we provided a novel regulatory mechanism for ANRIL in gastric cancer, in which ANRIL silence down-regulated BMI1 via miR-99a, along with activation of the apoptotic pathway and inhibition of the Notch and mTOR pathways.

## Introduction

Gastric cancer is one of the most common malignancies worldwide, ranking second in terms of global cancer related mortality (1). Currently, surgical resection therapy and chemotherapy have been practiced in patients with gastric cancer (2). However, gastric cancer is diagnosed at an advanced stage accompanied by malignant proliferation in most patients, with poor prognosis for advanced-stage patients (3). Currently, gastric cancer is still a big burden for health resources and facilities (4). Therefore, elucidating the molecular mechanisms underlying gastric carcinogenesis is essential for improving diagnosis and prognosis of gastric cancer.

The development of cancer is a complex process, in which multiple oncogenes and cancer suppressor genes are involved. For instance, microRNAs (miRNAs, miRs) and long non-coding RNAs (lncRNAs), including miR-148b, miR-10b, lncRNA H19 etc., have been identified as key factors for tumorigenesis of gastric cancer (5–7). lncRNAs are 200 nt-100 kb in length and do not have an obvious potential to code for a functional protein; they have been previously considered as “dark matter” of the transcriptome (8,9). Many lncRNAs are known to play important roles in cellular development, differentiation, and other processes (10–12). The function of lncRNAs in regulating gene expression during human cancer has been verified (13). In previous studies by Li et al. (14) in 2016 and Wan et al. (15) in 2015, lncRNAs have been shown to interact with DNA, RNA, and proteins, and thereby playing essential roles in gastric tumorigenesis by affecting cell cycle, migration and invasion, and apoptosis. Hence, lncRNAs become a hotspot for exploring therapeutic targets of the gastric cancer.

Antisense non-coding RNA in the INK4 locus (ANRIL) is a 3.8 kb lncRNA encoded in the chromosome 9p21 region and reported to be up-regulated in gastric cancer tissues (16,17). In addition, ANRIL knockdown could significantly up-regulate the expression of miR-99a/miR-449a both in SGC-7901 and BGC-823 cell lines in a polycomb repressive complex (PRC) 2-dependent manner (18). As a member of PRC1, B-lymphoma Mo-MLV insertion region 1 (BMI1) has been reported to be overexpressed in advanced stages and related to poor prognosis in many cancers (19). Therefore, we hypothesized that there might be a relationship among ANRIL, miR-99a, and BMI1 in gastric cancer, however, there is currently not enough literature on this subject. A previous study has reported the potential correlation between BMI1 and the Notch signaling cascade (20). Notch signaling promotes proliferative signaling and plays a major role in human tumor development including gastric cancer (21). Meanwhile, the mammalian target of rapamycin (mTOR) mainly functions through the PI3K/AKT/mTOR pathway to participate in regulation of cell growth and cell cycle and other physiological functions (22). Therefore, the alteration of these signaling cascades was also investigated.

In the present study, expression of ANRIL was measured in gastric cancer tissues and cell lines. We investigated the effect of ANRIL on miR-99a expression and their regulations of cell proliferation and apoptosis, as well as the expression of BMI1 *in vitro* by knockdown of ANRIL in MKN-45 and SGC-7901 cells. In addition, we demonstrated the effects of abnormally expressed BMI1 on apoptotic pathway and regulation of Notch and mTOR pathways, providing a rational explanation for ANRIL-mediated cell viability, migration, invasion, and apoptosis.

## Material and Methods

### Clinical sample collection

Twenty paired human gastric cancer tissues and the corresponding adjacent non-tumor tissues were obtained from patients who had undergone surgeries at the Affiliated Hospital of Qingdao University between 2014 and 2015. All patients with gastric cancer were diagnosed pathologically according to the criteria of the American Joint Committee on Cancer. None of the patients received any therapy before surgery. The study was approved by the local institutional ethics committee and written informed consent was obtained from every patient before specimen collection. All samples were immediately frozen in liquid nitrogen and stored until required.

### Cell culture

The human gastric epithelial cell line GES-1 and human gastric cancer cell lines MKN-45 and SGC-7901 were obtained from Institutes for Biological Sciences Cell Resource Center (China) and were cultured in high glucose Dulbecco's modified Eagle's medium (DMEM) supplemented with 10% fetal bovine serum (FBS; Gibco, USA). Cells were incubated at 37°C in a humidified incubator with 5% CO_2_. The exponentially growing cells were used.

### RNA isolation and quantitative real-time PCR (qPCR)

Total RNAs in cells or tissues were isolated using Trizol reagent (Invitrogen, USA) and the quality of RNA was evaluated according to the manufacturer's instructions. RNAs (500 ng) were reverse transcribed to cDNA using NCode miRNA First-Strand cDNA synthesis kit (Invitrogen). The expression levels of ANRIL in tissues and cells were measured by qPCR using One Step SYBR® PrimeScript^TM^ PLUS RT-RNA PCR kit (TaKaRa Biotechnology, China) according to the manufacturer's protocol, with normalization to GAPDH. Meanwhile, Taqman MicroRNA Reverse Transcription kit and Taqman Universal Master Mix II (Applied Biosystems, USA) were used for testing the expression levels of miR-99a, with normalization to U6 in cell lines. Primer sequences used in our study are shown in the Supplementary Table S1. All experiments were performed using the 2^-ΔΔCt^ method (23). Each experiment was repeated three times.

### Cell transfection

Cells were reseeded in 6-well plates and cultured for 24 h. Both MKN-45 and SGC-7901 cells were then transfected with recombinant expression vectors small hairpin RNAs (shRNAs) or miRNAs, respectively. The overexpression vector pEX-BMI1 and its negative control (empty pEX-2) were synthesized (Life Technologies, Invitrogen), the specific shRNA for ANRIL or BMI1 was cloned into pENTR^TM^/U6 vector (GenePharma, China), and the resultant plasmids were referred to as shANRIL and shBMI1, respectively. The pENTR^TM^/U6 vector carrying a non-targeting sequence, which was referred to as shNC, was purchased from GenePharma. For miR-transfection, the miR-99a mimic, inhibitor, and the scramble controls (mimic control and inhibitor control) were purchased from RiboBio Co., Ltd. (China). The nucleotide sequences are shown in the Supplementary Table S2. All transfections were performed using lipofectamine 3000 reagent (Invitrogen) according to the manufacturer's protocol. After 48 h of transfection, cells were collected for further analysis. The stably transfected cells were selected by the culture medium containing 0.5 mg/mL G418 (Sigma-Aldrich, USA) and the selection lasted for about 4 weeks.

### Cell viability assay

Cell viability was determined using the Cell Counting Kit-8 (CCK-8, Dojindo, Japan), according to the manufacturer's instructions. In brief, the MKN-45 and SGC-7901 cells were seeded in 96-well plates at 5×10^3^ cells/well and pre-cultured. After 48 h of transfection, 10 μL of CCK-8 solution was added to every well and the cells were incubated for another 1 h at 37°C in humidified atmosphere containing 95% air and 5% CO_2_. Absorbance was measured at 450 nm using a Microplate Reader (Bio-Rad, USA).

### Migration and invasion assay

Cell migration was determined by a modified two-chamber migration assay, with a chamber pore size of 8 μm (No. 662638, Greiner Bio-One GmbH, Germany). The cells were suspended in 200 μL of serum-free culture medium and seeded on the upper compartment of a 24-well Transwell culture chamber. For the lower compartment, 600 μL of complete medium was added. The chamber was incubated for 12 h at 37°C, and cells were fixed with methanol for 30 min at the end of culture. Non-traversed cells were carefully removed from the upper surface of the filter using a cotton swab. Traversed cells on the lower side of the filter were stained with 0.1% crystal violet (Amresco, USA) and counted under a microscope (Leica Microsystems, Germany). The protocol of cell invasion was the same as that of cell migration except for the filter being pre-coated with Matrigel (BD Biosciences, USA).

### Early apoptosis assay

After cells were treated by the indicated conditions, early apoptosis was detected by using annexin V-PE/7-amino-actinomycin D (7-AAD) cell apoptosis detection kit (BD Biosciences). Briefly, cells were washed in phosphate-buffered saline and stained in PE conjugated annexin V and 7-AAD for 15 min in the dark. The early apoptotic cells were detected by flow cytometry (BD Biosciences). The measurement was performed in triplicate and data were analyzed by FlowJo software (Tree Star, USA).

### Western blotting assay

Cellular proteins were extracted using RIPA lysis buffer (Beyotime Biotechnology, China) supplemented with protease inhibitors (Roche, USA), and then quantified using the BCA^TM^ protein assay kit (Pierce, USA). Proteins were separated using a Bis-Tris gel system (Bio-Rad) according to the manufacturer's instructions and then transferred onto the polyvinylidene difluoride (PVDF) membranes. Primary antibodies at a dilution of 1:1000 were incubated with membranes at 4°C overnight, followed by rinsing and incubation with secondary antibodies marked by horseradish peroxidase (1:5000; Santa Cruz Biotechnology, USA) for 1 h at room temperature. The membranes were transferred into the ChemiDoc^TM^ XRS system (Bio-Rad), and Immobilon Western Chemiluminescent HRP Substrate (Merck Millipore, USA) was added. The signals were captured using Image Lab^TM^ software (Bio-Rad). The primary antibodies used in this study were as follows: BMI1 (sc-10745); multiple tumor suppressor 1 (p16; sc-166760); B-cell lymphoma 2 (Bcl-2; sc-7328); caspase-3 (sc-271759); cleaved caspase-3 (sc-22171); caspase-9 (sc-56076); cleaved caspase-9 (sc-22182); Notch 1 (sc-376403); mTOR (sc-293089); phosphorylated mTOR (p-mTOR; s-c29313); p70 ribosomal protein S6 kinase (p70S6K; sc-9027), and phosphorylated p70S6K (p-p70S6K; sc-8416, all from Santa Cruz Biotechnology).

### Statistical analysis

All experiments were repeated three times. The results of multiple experiments are reported as means±SD. Statistical analyses were performed using GraphPad Prism 6 software (GraphPad Software, USA). The P values were calculated using one-way analysis of variance (ANOVA) for analysis between three or more groups or two-tailed Student's *t*-test for analysis between two groups (24). P<0.05 was considered to indicate a statistically significant result.

## Results

### ANRIL was up-regulated in human gastric cancer tissues and cells

**Figure 1.**
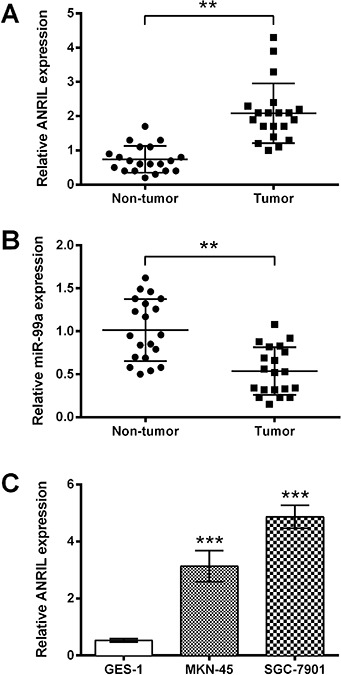
Antisense non-coding RNA in the INK4 locus (ANRIL) was up-regulated in gastric cancer. The levels of ANRIL and miR-99a were measured by qPCR. *A*, Expression of ANRIL and *B*, of miR-99a in gastric tumor tissues and adjacent non-tumor tissues (n=20). *C*, Expression of ANRIL in human gastric epithelial cell line GES-1 and human gastric cancer cell lines MKN-45 and SGC-7901. GAPDH acted as an internal control. Data are reported as means±SD. **P<0.01 (one-way ANOVA); ***P<0.001 (two-tailed Student's *t*-test).

The expression of ANRIL and miR-99a in gastric tumors or adjacent non-tumorous tissues as well as ANRIL expression in gastric epithelial cells or gastric cancer cells was detected by qPCR. The results in Figure 1 show that the ANRIL expression in gastric tumor tissues was higher than that in non-tumorous tissues (P<0.01, Figure 1A). However, the miR-99a expression in gastric tumor tissues was significantly lower than that in non-tumorous tissues (P<0.01, Figure 1B). Meanwhile, ANRIL level in gastric cancer cells (MKN-45 and SGC-7901 cells) was higher than that in normal gastric epithelial GES-1 cells (P<0.001, Figure 1C). These results suggested that the expression of ANRIL was up-regulated in both gastric tumor tissues and cell lines.

### Knockdown of ANRIL inhibited cell viability, migration, and invasion and promoted apoptosis

**Figure 2.**
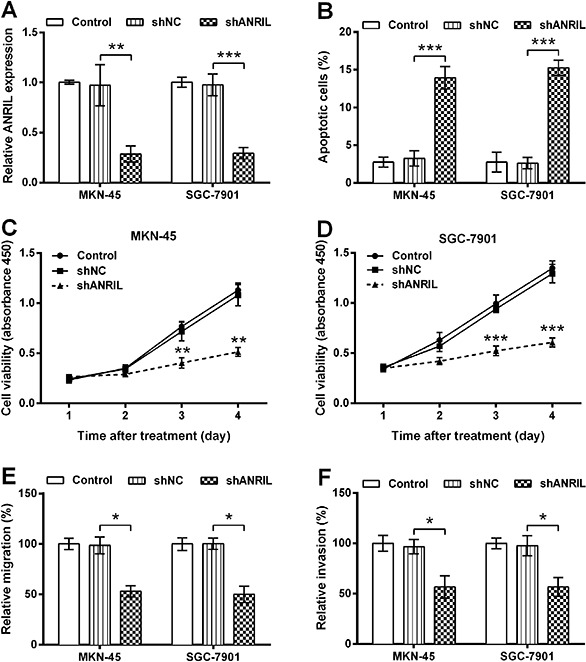
Knockdown of antisense non-coding RNA in the INK4 locus (ANRIL) inhibited cell viability, migration, and invasion, and promoted apoptosis. Untreated cells acted as control. *A*, Transfection efficiency in MKN-45 and SGC-7901 cells tested by qPCR. GAPDH acted as an internal control. *B*, Rate of apoptotic cells detected by flow cytometry. *C* and *D*, Cell viability determined by CCK-8 assay. The migration (*E*) and invasion (*F*) were measured by Transwell assay. shANRIL: pENTR^TM^/U6 vector carrying small hairpin RNA targeting ANRIL; shNC: pENTR^TM^/U6 vector carrying a non-targeting sequence; qPCR, quantitative real-time PCR; CCK-8, Cell Counting Kit-8. Data are reported as means±SD.*P<0.05; **P<0.01; ***P<0.001 (two-tailed Student's *t*-test).

To detect the effects of ANRIL in gastric cancer cells, ANRIL was knocked down using shANRIL. The transfection efficiency was detected by qPCR, and results are reported in Figure 2A. In both MKN-45 and SGC-7901 cells, the expression of ANRIL was significantly decreased after shANRIL transfection compared to the shNC group (P<0.01 or P<0.001). This suggested that shANRIL transfection could effectively down-regulate ANRIL expression in MKN-45 and SGC-7901 cells.

The results in Figure 2B showed that knockdown of ANRIL could significantly increase the rate of apoptotic cells in both MKN-45 and SGC-7901 cells compared to the shNC group (both P<0.001). The CCK-8 assay results showed that the cell viability was significantly reduced by ANRIL knockdown at 3 and 4 d post-transfection compared with the shNC groups in MKN-45 and SGC-7901 cells (P<0.01 or P<0.001, Figure 2C and D). Migration and invasion of MKN-45 and SGC-7901 cells were significantly inhibited after ANRIL knockdown as compared to the shNC groups (all P<0.05, Figure 2E and F). These results suggested that ANRIL suppression might be defective in gastric cancer.

### ANRIL regulated the expression of miR-99a in gastric cancer cells

**Figure 3.**
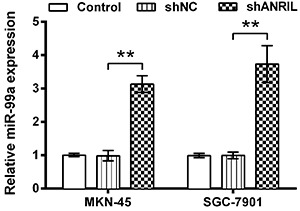
Knockdown of antisense non-coding RNA in the INK4 locus (ANRIL) up-regulated miR-99a expression. Untreated cells acted as control. The expression level of miR-99a was measured by qPCR. U6 acted as an internal control. shANRIL: pENTRTM/U6 vector carrying small hairpin RNA targeting ANRIL; shNC: pENTRTM/U6 vector carrying a non-targeting sequence; qPCR, quantitative real-time PCR; miR-99a, microRNA-99a; Data are reported as means±SD.**P<0.01 (two-tailed Student's *t*-test).

There is a report showing that ANRIL expression is inversely correlated with miR-99a expression in gastric cancer tissues (25). In this study, we detected the relationship between ANRIL and miR-99a expression in MKN-45 and SGC-7901 cells. After cell transfection, the expression of miR-99a in cells transfected with shANRIL was significantly higher than that of the shNC group in MKN-45 and SGC-7901 cells (both P<0.01, Figure 3). It suggested that the expression of miR-99a was negatively related with ANRIL expression in both MKN-45 and SGC-7901 cells.

**Figure 4.**
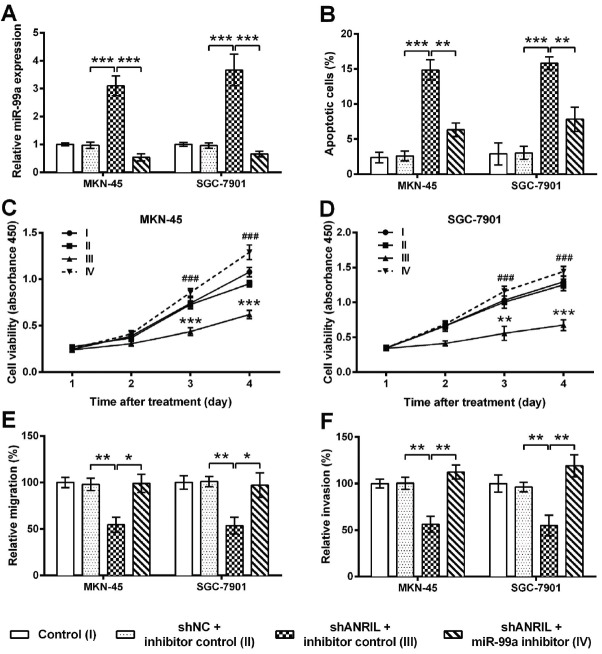
Increase of cell apoptosis and decreases of cell viability, migration, and invasion induced by knockdown of antisense non-coding RNA in the INK4 locus (ANRIL), were reversed by miR-99a inhibition. Non-treated cells acted as control. *A*, Expression level of miR-99a measured by qPCR. U6 acted as an internal control. *B*, Rate of apoptotic cells by flow cytometry. *C* and *D*, Cell viability was determined by CCK-8 assay. The migration (*E*) and invasion (*F*) were measured by Transwell assay. shANRIL: pENTR^TM^/U6 vector carrying small hairpin RNA targeting ANRIL; shNC: pENTR^TM^/U6 vector carrying a non-targeting sequence; qPCR: quantitative real-time PCR; CCK-8: Cell Counting Kit-8; miR-99a: microRNA-99a; Data are reported as means±SD. *P<0.05; **P<0.01; ***P<0.001; ^###^P<0.001 (two-tailed Student's *t*-test). In panels *C* and *D*, *indicates significant difference between the shNC + inhibitor control group and the shANRIL + inhibitor control group, and ^#^indicates significant difference between the shANRIL + inhibitor control group and the shANRIL + miR-99a inhibitor group.

Then, stably transfected cells were transfected with inhibitor control or miR-99a inhibitor for exploring whether ANRIL affected gastric cancer cells through modulation of miR-99a. Results in Figure 4A show that ANRIL silence-induced up-regulation of miR-99a was significantly reversed by miR-99a inhibitor (both P<0.001). Meanwhile, increase of cell apoptosis (Figure 4B) and decreases of cell viability (Figure 4C and D), migration (Figure 4E), and invasion (Figure 4F), which were induced by ANRIL knockdown, were all reversed by miR-99a inhibition (P<0.05, P<0.01, or P<0.001). These results suggested that ANRIL might affect gastric cancer cells by regulating miR-99a expression.

### ANRIL knockdown down-regulated the expression of BMI1 by modulating miR-99a in gastric cancer cells *in vitro*

**Figure 5.**
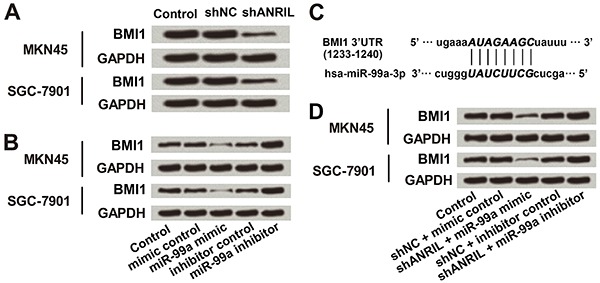
Knockdown of antisense non-coding RNA in the INK4 locus (ANRIL) inhibited the expression of BMI1 via miR-99a up-regulation. Protein expressions of BMI1 were measured by western blotting. *A*, ANRIL silence down-regulated BMI1 expression. *B*, Expression of BMI1 was negatively correlated with miR-99a expression. *C*, Putative binding sequence between miR-99a-3p and BMI1 3′UTR using TargetScan software. *D*, Down-regulation of BMI1, induced by ANRIL knockdown, was reversed by miR-99a inhibition. shANRIL: pENTR^TM^/U6 vector carrying small hairpin RNA targeting ANRIL; shNC: pENTR^TM^/U6 vector carrying a non-targeting sequence; miR-99a: microRNA-99a; BMI1: B-lymphoma Mo-MLV insertion region 1; 3′UTR: 3′-untranslated region.

As shown in Figure 5A, knockdown of ANRIL inhibited the expression of BMI1 in MKN-45 and SGC-7901 cells. BMI1 was down-regulated by miR-99a mimic but was up-regulated by miR-99a inhibitor (Figure 5B). The putative binding sequence between miR-99a-3p and BMI1 is shown in Figure 5C. Further experiments showed that combination of ANRIL silence and miR-99a overexpression down-regulated BMI1 expression, whereas BMI1 level in cells transfected with shANRIL and miR-99a inhibitor was nearly the same as that of the shNC + mimic control groups (Figure 5D). These results suggested that knockdown of ANRIL down-regulated the expression of BMI1 via up-regulation of miR-99a.

### BMI1 inhibited apoptotic related pathway in MKN-45 and SGC-7901 cells

**Figure 6.**
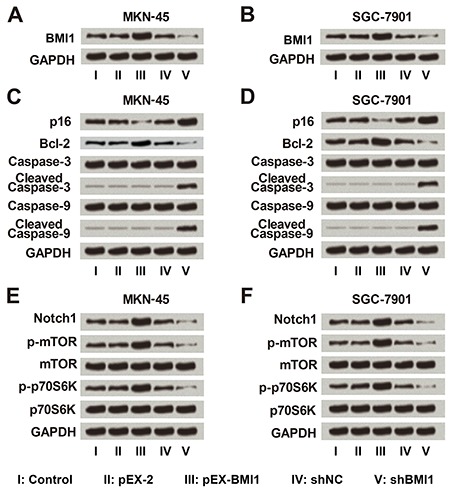
BMI1 inhibited the apoptotic pathway and activated the Notch and mTOR pathways. Protein expression was determined by western blotting. *A* and *B*, BMI1 was abnormally expressed after cell transfection. *C* and *D*, Bcl-2 expression was down-regulated while expressions of p16, cleaved caspase-9, and cleaved caspase-3 were up-regulated by BMI1 silence. *E* and *F*, Phosphorylated levels of key kinases in the Notch and mTOR pathways were increased by BMI1 overexpression. BMI1: B-lymphoma Mo-MLV insertion region 1; pEX-BMI1: recombined overexpression vector of BMI1; shBMI1: pENTR^TM^/U6 vector carrying small hairpin RNA targeting BMI1; shNC: pENTR^TM^/U6 vector carrying a non-targeting sequence; p16: multiple tumor suppressor 1; Bcl-2: B-cell lymphoma 2; mTOR: mammalian target of rapamycin; p70S6K: p70 ribosomal protein S6 kinase; p-: phosphorylated.

Next, we explored the regulation of BMI1 expression on related pathways. As shown in Figure 6A and B, BMI1 was overexpressed in cells transfected with pEX-BMI1, and the expression of BMI1 was decreased after transfection with shBMI1 in both MKN-45 and SGC-7901 cells. The expression of p16 was inhibited after BMI1 overexpression and increased after knockdown of BMI1 (Figure 6C and D). The expression of Bcl-2 showed the opposite trend in both types of cells. The expression of cleaved caspase-3 and cleaved caspase-9 was increased in BMI1-silenced cells. These results suggested that expression of BMI1 affected the expression of apoptosis-related proteins and thus inhibited the activation of the apoptotic pathway.

### BMI1 activated Notch and mTOR signal pathways

The BMI1 was overexpressed or knocked down in MKN-45 and SGC-7901 cells, respectively. As shown in Figure 6E and F, the expression of Notch1 was increased after overexpression of BMI1 and decreased in BMI1-silenced cells. The same expression trends were shown in phosphorylated mTOR and p70S6K in both MKN-45 and SGC-7901 cells. Accordingly, we speculated that overexpression of BMI1 could activate the Notch and mTOR signaling pathways.

## Discussion

Gastric cancer is the most common gastrointestinal malignancy and the curative effect of existing treatments is still limited. Therefore, new biomarkers and therapeutic targets for gastric cancer are urgently needed (26). Increasing evidence demonstrates that mammalian genomes encode thousands of lncRNAs, which might display a large regulatory component of the eukaryotic genome (27). Recent research has demonstrated that dysregulation of lncRNAs regulates human diseases including tumors (28). In this study, we identified the higher expression of lncRNA ANRIL in gastric cancer tissues than in corresponding non-tumor tissues, and it was also up-regulated in MKN-45 and SGC-7901 cells compared with gastric epithelial cell GES-1 *in vitro*, indicating that the abnormal expression of ANRIL in gastric cancer could be a candidate biomarker in gastric cancer diagnosis.

ANRIL belongs to the family of lncRNAs, which have been implicated in diverse functions of gene regulation that is mediated by RNA-RNA, RNA-DNA, or RNA-protein interactions (29,30). A previous study has shown that ANRIL is associated with tumor size and advanced TNM stage in gastric cancer patients, and high ANRIL expression in gastric cancer tissues is associated with a poor prognosis (25). Therefore, we assessed the effects of abnormally expressed ANRIL on gastric cancer cells *in vitro*. Consistent with previous studies (31,32), we found that ANRIL knockdown could significantly inhibit gastric cancer cell viability, migration, and invasion, and promoted apoptosis in MKN-45 and SGC-7901 cells *in vitro*, consolidating that ANRIL played an important role in gastric cancer progression.

lncRNAs and miRNAs are both important non-coding RNAs in eukaryotes implicated in organism development and in various human diseases; however, little is known about the relationship between them (33). Recent studies showed that lncRNAs could function as a ‘sponge’ to titrate miRNAs (34). Our results showed that knockdown of ANRIL significantly up-regulated the expression of miR-99a, and more importantly, it indicated that ANRIL could crosstalk with miR-99a to regulate gastric cell viability, migration, invasion, and apoptosis.

ANRIL has been postulated to be a scaffold for the PRC1 and PRC2 (35). As BMI1 is a member of PRC1, we supposed that there might be a relationship between ANRIL and BMI1. The experiments showed that ANRIL knockdown decreased BMI1 expression. Then, after abnormal expression of miR-99a, BMI1 expression was negatively correlated with miR-99a expression. In addition, ANRIL silence-induced down-regulation of BMI1 could be abrogated by miR-99a inhibition, suggesting that ANRIL knockdown decreases BMI1 expression through up-regulating miR-99a. A previous study confirmed that BMI1 plays an important role in maintaining the proliferation of cells, and BMI1 suppression could promote apoptosis (36). In our study, BMI1 silence up-regulated the expression of p16, which is also known as multiple tumor suppressor 1, and down-regulated the expression of Bcl-2, followed by up-regulations of cleaved caspase-3 and cleaved caspase-9, indicating that BMI1 silence could activate the apoptotic pathway in MKN-45 and SGC-7901cells. As a novel antitumor gene, p16 expression has great clinical significance in predicting tumor prognosis (37). Alterations of p16 after abnormal expression of BMI1, which is regulated by ANRIL via modulating miR-99a, indicated that ANRIL might be a prognostic marker for gastric cancer.

In addition, the Notch signaling pathway is a highly conserved cell signaling system present in most multicellular organisms (38). It has been verified that abnormal Notch1 plays an important role in regulation of tumor cell proliferation and apoptosis (39). Meanwhile, mTOR also plays a pivotal role in cell growth and cell cycle regulation as well as other physiological functions (22). Results in this study showed that ANRIL could activate the Notch and mTOR pathways through miR-99a-mediated modulation of BMI1 in MKN-45 and SGC-7901 cells, thus regulating cell viability, migration, invasion, and apoptosis.

In conclusion, this study found that lncRNA ANRIL was up-regulated in gastric cancer and its knockdown could crosstalk with miR-99a, reducing cell viability, migration, and invasion while increasing cell apoptosis in gastric cancer cells *in vitro*. Importantly, we provided a novel regulatory mechanism of ANRIL in gastric cancer, by which ANRIL functioned through miR-99a-mediated modulation of BMI1, involved in the apoptotic pathway, and in Notch and mTOR signal pathways. We hope these results might facilitate the development and use of lncRNA in diagnostics and therapeutics of gastric cancer. Moreover, ANRIL silence, miR-99a up-regulation, and BMI1 silence might be potential therapeutic strategies for gastric cancer.

## Supplementary Material

Click here to view [pdf].
